# Long-term outcome after adenotonsillectomy in Down Syndrome with OSAS

**DOI:** 10.1016/j.bjorl.2026.101883

**Published:** 2026-09-02

**Authors:** Sara Costa Gomes, Jaouad Abari, Helen Franckx, Marie Reynders, Viki Vanderstraeten, Linde Peeters, Wilfried Cools, Ina Foulon

**Affiliations:** a Universiteit Gent (UGent), University Hospital of Ghent (UZ Gent), Department of Otorhinolaryngology, Ghent, Belgium; b Vrije Universiteit Brussel (VUB), University Hospital of Brussels (UZ Brussel), Department of Otorhinolaryngology Head and Neck Surgery, Brussels, Belgium; c University of São Paulo (USP), Department of Otorhinolaryngology and Ophthalmology, São Paulo, SP, Brazil; d Vrije Universiteit Brussel (VUB), University Hospital of Brussels (UZ Brussel), Department of Pediatrics, Pediatric Neurology Unit, Brussels, Belgium; e Vrije Universiteit Brussel (VUB), University Hospital UZ Brussel (UZ Brussel), Department of Pediatric Pneumology, Brussels, Belgium; f Vrije Universiteit Brussel (VUB), UZ Brussel, UMC Onderzoeksmanagement Dienst, Research Management Service, Brussels, Belgium

**Keywords:** Adenotonsillectomy, Down syndrome, Pediatrics, Polysomnography, Sleep apnea

## Abstract

**Objectives:**

The study aimed to evaluate short- and long-term polysomnographic parameters in children with Down Syndrome (DS) and Obstructive Sleep Apnea Syndrome (OSAS) submitted to surgical therapy. Secondarily, this study also aimed to describe how other congenital comorbidities influence sleep patterns and to present trends in Body Mass Index (BMI) percentile/Z-score after adenotonsillectomy in DS population.

**Methods:**

Longitudinal retrospective-cohort study of children with Down syndrome and respiratory sleep disorders. Patients with habitual snoring that underwent polysomnographic exams and surgical therapy were included (n = 54). We excluded patients >18-years old by the time of the surgery and who presented diagnostic polysomnography with Total Sleep Time (TST) <180 min, ending in a group of 25 children. The post-therapy parameters were divided in short- (<18-months) and long-term (>18-months) results, as heterogeneity in follow-up intervals occurred.

**Results:**

Of the 25 subjects, the short- and long-term Obstructive Apnea-Hypopnea Index (OAHI) were both significantly different from the pre-surgery value. However, there is also a difference between the short- and long-term OAHI post-surgery (p < 0.001). Children with laryngomalacia present the highest OAHI mean pre-surgery (56.7 ± 13.8).

**Conclusions:**

Adeno/tonsillectomy for children with DS and OSAS brings a significant lowering on OAHI in a short- and long-term post-surgical follow-up, although long-term OAHI is significantly higher than the short-term. An extended polysomnographic post-surgical follow-up should be recommended. Due to the study limitations, further studies are still needed to clarify the best practice for this specific population.

**Level of evidence:**

Level 3.

**Clinical trial registration:**

Not applicable.

## Highlights

• Adenotonsillectomy decreases OAHI in a short- and long-term post-surgical follow-up.
• Long-term OAHI is significantly higher than the short-term.
• 28% of children (short-term) and 44% (long-term post-surgery) remained with OSAS.
• Central apnea index increases in the short- and long-term post-surgery periods.
• The association among comorbidities and OAHI showed no statistical significance.

## Introduction

Down's Syndrome (DS) is the most common genetic disorder. With an incidence of approximately 1:1000 live born infants,^1^^,^^2^ this disorder reaches estimated numbers of 200,000 individuals in Europe and 2 million worldwide.^3^^,^^4^ Among a variety of medical conditions, the Obstructive Sleep Apnea Syndrome (OSAS) is one of the most common in these patients and may prevent them to reach their full developmental capacity.^1^^,^^2^ In children with DS, OSAS prevalence varies from 57% to 82.3%.^1^^,^^5^^,^^6^ The cause for this high prevalence relies on DS craniofacial-dysmorphologies,^2^^,^^7^^,^^8^ besides the adenotonsilair hypertrophy. Although Adenotonsillectomy (AT) is the treatment of choice for OSAS in children with adenotonsillar hyperplasia and leads at least temporarily to the resolution of this condition in about 79% of the general pediatric population, positive results from this surgery in children with trisomy 21 ranges from 6% to 33%.^9^

Polysomnography (PSG) is the gold-standard exam for the diagnosis of OSAS.^2^ This exam helps in decision making for surgery indication, as it defines OSAS severity and the need for a more extensive postoperative monitoring. Considering that the surgery is not curative for all the cases, postoperative PSG is also indicated to identify residual OSAS with need of further treatment.^6^^,^^10^ A knowledge gap concerning the long-term polysomnographic post-operative follow-up of this population remains. This study primary objective was to evaluate short-(<18-months) and long-term (>18-months) polysomnographic parameters in children with DS and Sleep-Disordered Breathing (SDB) submitted to surgical therapies. Secondarily, this study also aimed to describe how congenital comorbidities and BMI percentile/Z-score influence sleep patterns.

## Methods

Longitudinal retrospective cohort study of children with DS and SDB with sleep-therapy carried out from 2009 and 2024. Patients with DS who underwent surgery for OSAS under 18-years old, by the date of the surgery, were selected. We excluded patients with a diagnostic PSG Total Sleep Time (TST) < 180 min and who lack both pre- and post-surgery PSG. The surgical indication depended on the nasopharynx obstruction level in the nasofibroscopy exam or after cavum palpation under sedation (> 50% obstruction), and the oropharynx obstruction level (≥ Grade II). Polysomnographic exams were requested pre- and post-operatively, depending on the date of patient’s return for consultations and on the feasibility of the exam for this population.

Sleep parameters were evaluated according to the criteria of the American Academy of Sleep Medicine.^11^ The diagnosis for OSAS is based on both clinical and polysomnographic criteria. The polysomnographic criteria are more than one apnea per hour of sleep (obstructive, mixed or hypopneas) or hypercarbia >50 mmHg >25% of the total sleep time with at least snoring or flattening of inspiratory nasal pressure waveform or paradoxical thoraco-abdominal motion. Pediatric diagnosis of OSAS was defined by the Obstructive Apnea-Hypopnea Index (OAHI) ≥1.0 events/hour and a minimum percutaneous oxygen saturation value <92%. As for the severity, it is considered cured/resolved OSAS as OAHI < 1, mild <5, moderate <10, and severe ≥10.^11^, ^12^, ^13^, ^14^ This project was approved by the institutional ethics committee, under number EC-2024-193. Data analysis was performed after codification and pseudonymization of patients’ profiles, to keep the confidentiality and to preserve data safety.

The therapy based on surgical intervention for OSAS was represented mainly by adenotonsillectomy under orotracheal intubation and general anesthesia. The surgical indication depended on the nasopharynx obstruction level in the nasofibroscopy exam or after cavum palpation under sedation. Besides that, the combination of Mallampati score with tonsil-hypertrophy grade played an important role for the joint surgical decision with the parents. The tonsillectomy technique used was tonsil dissection. The adenoidectomy was performed via adenoid curettes. Specific aspects of other clinical therapies, such as sleep hygiene measurements, were not described in this study.

Data was collected as follows: age, gender, surgery ‒ adenoidectomy and/or tonsillectomy, associated ventilation ear tubes, associated therapy with Continuous Positive Airway Pressure (CPAP), comorbidities (hypothyroidism, laryngomalacy, heart diseases as Atrial Septal Defect (ASD) / Atrioventricular Septal Defect (AVSD) / Ventricular Septal Defect (VSD), diabetes mellitus, Hirschsprung disease, subglotic stenosis, lip/palate cleft, Partial agenesis corpus callosum, Fallot’s tetralogy, omphacele, West syndrome) and pre- and post-therapy polysomnographic parameters: weight, height, Body Mass Index (BMI), BMI percentile, *Z*-Score, OSAS severity, TST, Rapid Eye Movement stage (REM, %), Apnea Hypopnea Index ‒ AHI (n/h), AHI Non-Rapid Eye Movement stage (NREM, n/h), AHI-REM (n/h), OAHI (n/h), Obstructive Apnea Index ‒ OAI (n/h), Central Apnea Index ‒ CAI (n/h), Mixed Apnea Index – MAI (n/h), Arousal Index ‒ AI (n/h), Minimum Oxygen Saturation ‒ MinO_2_Sat (%), percutaneous oxyhemoglobin saturation-SpO_2_< 90% sleep-time (%). The percentage is in reference to total sleep time. ENT comorbidities were checked by fibroscopy and/or imaging, whereas other comorbidities by imaging and blood test. The polysomnographic exams were interpreted by only one sleep specialist. PSG parameters were classified in pre-surgery, short-term (<18-months) and long-term (>18-months) post-surgery.

### Statistical analysis

Statistical analysis was performed with *R* Core Team (2021) Foundation for Statistical Computing, Vienna, Austria. OAHI values pre, short- and long-term post-surgery were modelled with a Linear Mixed Model which accounts for the correlations induced by the repeated measurements. After the model was fit, Tukey post hoc comparisons were applied. Data is reported in results as mean ± standard deviation [minimum – maximum values]. Missing data was taken into account during statistical analysis.

## Results

The electronic patients’ files from 54 patients were identified from the hospital database. After applying the exclusion criteria, the final group consisted of 25 patients. Sixteen patients were male, mean age at the time of surgery 4.28 ± 3.41 [1–13]. There were 16 (64%) adenotonsillectomy cases, 6 (24%) adenoidectomy (alone), and 3 (12%) tonsillectomy (alone) cases. There were 17 (68%) children that underwent polysomnography pre-surgery and 19 (76%) children that underwent PSG post-surgery. Out of this, 12 PSG were short-term post-surgery and 12 PSG were long-term post-surgery, as some of these children underwent both short and long-term PSG. Five patients had both short and long-term post-surgery PSG, and 7 patients had only long-term PSG. Short-term post-surgery PSG were performed within a mean interval of 3.25 ± 4.35 [1–13] months, and long-term post-surgery PSG 53.25 ± 33.95 [18–111] months. As the age at initial PSG can have influence in the surgery outcomes, this study analyzed the age of the subjects at each PSG. The mean age of the subjects at PSG pre-surgery was 3.94 ± 3.45 [1–13], at PSG post < 18-months was 5.17 ± 3.48 [2–13] and at PSG post >18-months was 8.83 ± 4.18 [4–17]. Concerning the CPAP, 3 subjects underwent CPAP therapy prior to the surgery and 1 subject started after surgery (4-years after the procedure).

**Fig. 1:**
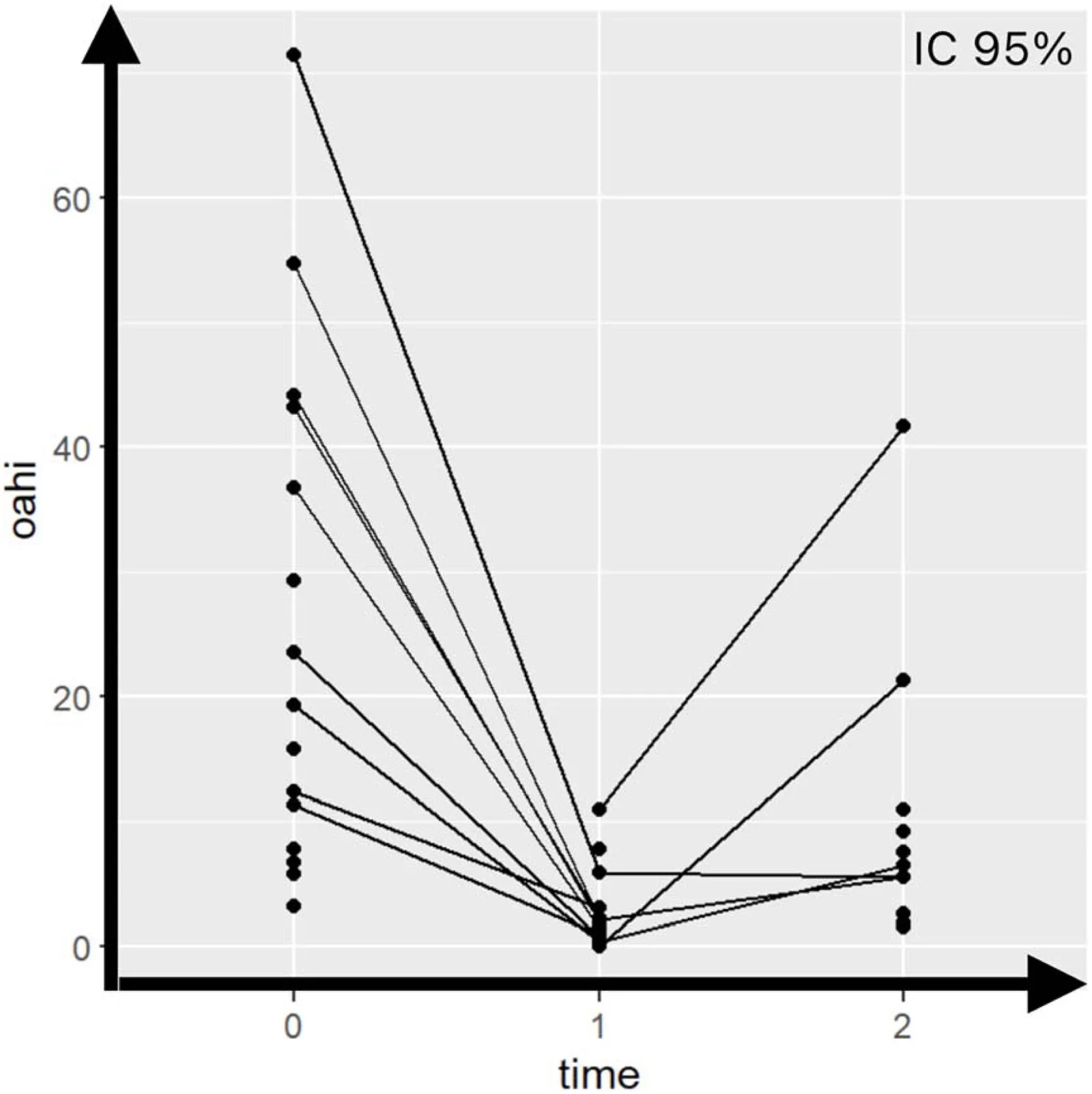
OAHI over time: pre-surgery, short- and long-term post-surgery. 0 = Pre-surgery, 1 = short-term post-surgery, < 18 months, 2 = long-term post-surgery, > 18 months. There are significant differences between pre-surgery and post-surgery <18 m (24.804, p < 0.001), between pre-surgery and post-surgery >18 m (15.367, p = 0.01), and between post-surgery <18 m and post-surgery >18 m (9.437, p < 0.001). 17 children underwent PSG pre-surgery (time 0) and 19 children underwent PSG post-surgery (time 1 and/or time 2), being 12 PSG time 1 and 12 PSG time 2 (95% IC).

The linear mixed model combined all data to explore the changes over time. The analysis addressing 25 patients showed a decrease in OAHI for especially patients that start highest at the pre-surgery, compared to both the short-term (difference = -24.804, p < 0.001) and long-term post-surgery (difference = -15.367, p = 0.01). However, there is also evidence that there is a difference between the short- and long-term post-surgery groups (difference = 9.437, p < 0.001), see Fig. 1. Out of these 25 children, 28% in a short-term and 44% in a long-term post-surgery remained with residual OSAS. Pre-operatively, there were 12 severe cases, 3 moderate and 2 mild cases, OAHI 25.55 ± 19.70 [3.2–71.4]. Post-operatively short-term, there were 0 severe cases, 2 moderate, 5 mild and 4 resolved cases, OAHI 3.2 ± 3.38 [0–11.0]. Post-operatively long-term, there were 2 severe cases, 6 moderate, 3 mild and 1 resolved case, OAHI 10.37 ± 11.76 [1.5–41.7]. See Table 1 for the evolution of OSAS over time. Regarding the comparison pre and post < 18-months, there is a decrease in the values of N1 sleep stage and an increase in N3, a deeper stage. Besides that, TST values increased, the arousal index significantly decreased and the percentage of sleep time with SpO_2_ <90% decreased. An improvement in oxygenation was also noticed, with T90 decreasing from 10% to 2.9% (at <18-months) and 1.7% (at >18-months). As for the minimum oxygen saturation, the study showed an unexpected decrease in post <18-months with a subsequent increase in post >18-months. Central apnea index also presents a similar paradoxical presentation, as an increase of central apneas was demonstrated in a short- and in a long-term post-surgery, when compared to pre-surgery values. See Table 2 for the analysis of further polysomnographic parameters.

**Table 1:** Evolution of OSAS over time.

	OSAS post-op <18-months			
OSAS pre-op	Resolved	Mild	Moderate	No PSG
Mild	1	0	0	1
Moderate	0	0	0	3
Severe	3	5	1	3
No PSG	0	0	1	7

	OSAS post-op >18 months				
OSAS post-op <18 months	Resolved	Mild	Moderate	Severe	No PSG
Resolved	0	0	2	0	2
Mild	0	0	1	0	4
Moderate	0	0	1	0	1
No PSG	1	3	2	2	6

Pre-op, Pre-operative; Post-op, Post-operative.

**Table 2:** Evolution of polysomnographic parameters of patients with OSAS.

	Pre	Post <18-months	Post >18-months
Weight (kg)	20.19 ± 18.24	26.51 ± 23.69	38.51 ± 29.25
Height (cm)	95.90 ± 24.0	106.85 ± 26.21	120.12 ± 21.46
BMI	18.80 ± 5.99	19.64 ± 7.21	23.29 ± 9.50
TST (minutes)	430.12 ± 72.69	449.17 ± 74.44	451.50 ± 119.15
REM (%)	23.77 ± 12.63	20.99 ± 11.30	20.24 ± 12.74
AHI (n/h)	22.88 ± 15.33	3.42 ± 2.94	10.39 ± 10.82
OAHI (n/h)	25.55 ± 19.70	3.12 ± 3.38	10.37 ± 11.76
OAI (n/h)	6.31 ± 6.89	0.66 ± 1.09	3.24 ± 6.75
CAI (n/h)	1.44 ± 4.77	24.82 ± 24.07	8.28 ± 16.96
MAI (n/h)	0.69 ± 2.75	2.58 ± 6.64	1.09 ± 3.62
AI (n/h)	26.48 ± 13.10	12.83 ± 4.73	14.80 ± 7.27
MinO2Sat (%)	75.29 ± 12.62	72.03 ± 16.82	81.61 ± 12.12
AHI NREM (n/h)	18.79 ± 18.55	2.22 ± 3.1	7.45 ± 11.41
AHI REM (n/h)	38.19 ± 26.16	7.26 ± 6.96	18.81 ± 18.78
SpO_2_<90% sleep time (%)	10.35 ± 16.52	2.93 ± 3.13	1.77 ± 2.87

Data expressed as mean ± standard deviation. BMI, Body Mass Index; TST, Total Sleep Time; REM, Rapid Eye Movement stage, REM stage as percentage of total sleep time; AHI, Apnea-Hypopnea Index; OAHI, Obstructive Apnea-Hypopnea Index; OAI, Obstructive Apnea Index; CAI, Central Apnea Index; MAI, Mixed Apnea Index; AI, Arousal Index; MinO_2_Sat, Minimum Oxygen Saturation; NREM, Non-Rapid Eye Movement; SpO_2_, Percutaneous Oxyhemoglobin Saturation.

Regarding the comorbidities, 11 out 25 (44%) presented at least one type of heart comorbidity. The most common congenital heart disease was ASD with 5 patients (20%), followed by AVSD with 4 patients (16%), and VSD with 2 (8%) patients. Children with laryngomalacia presented with the highest OAHI mean pre surgery (56.7 ± 13.8), followed by ASD (44.7 ± 23.9) and AVSD comorbidity (19.5 ± 12.2). The subjects with laryngomalacia were 3 out 25 (12%) in 2-, 3- and 4-years of age by the surgery time. Two out of these 3 also presented atrial septal defect as comorbidity. There was no description of associated development of pulmonary hypertension. These 3 subjects presented severe OSAS before surgery and all of them underwent adenotonsillectomy. Two out 3 achieved mild and 1 moderate OSAS by the time 2-months after surgery. Table 3 shows the frequencies of genders, comorbidities and performed surgeries, correlated to their mean OAHI over time. What concerns the increase of central apnea index post-surgery, it occurred explicitly in 7 subjects in a short-term, whereas 3 presented a heart disease (1 subject with associated laryngomalacia and 1 with associated hypothyroidism), and in 3 subjects in a long-term, all 3 presenting a heart disease (1 subject with associated laryngomalacia, 1 with associated hypothyroidism and 1 with associated Diabetes mellitus and Tetralogy of Fallot).

**Table 3:** Evolution of OAHI per gender, comorbidity and per type of performed surgery.

	Pre surgery OAHI	Post surgery OAHI <18 m	Post surgery OAHI >18 m
Gender			
Female (n = 9)	33.2 ± 18.8	3.16 ± 4.5	13.6 ± 13.9
Male (n = 16)	23 ± 20.1	3.1 ± 2.73	4.8 ± 3.32
Laryngomalacia			
Yes (n = 3)	56.7 ± 13.8	3.3 ± 2.25	5.5 ± 0
No (n = 22)	18.4 ± 12.4	3.07 ± 3.8	11.5 ± 12.9
ASD			
Yes (n = 5)	44.7 ± 23.9	2.35 ± 2.52	10.8 ± 9.12
No (n = 20)	19.2 ± 14.0	3.51 ± 3.84	10.2 ± 13.2
VSD			
Yes (n = 2)	19.3 ± NA	0.4 ± NA	7.85 ± 1.91
No (n = 23)	26.0 ± 20.3	3.37 ± 3.43	10.9 ± 13.1
AVSD			
Yes (n = 4)	19.5 ± 12.2	NA	7.5 ± NA
No (n = 21)	26.9 ± 21.2	3.12 ± 3.38	10.7 ± 12.4
Hypothyroidism			
Yes (n = 3)	19.3 ± NA	0.4 ± NA	8.7 ± 3.11
No (n = 22)	26.0 ± 20.3	3.37 ± 3.43	10.7 ± 13.1
Hirschsprung			
Yes (n = 2)	13.4 ± 14.4	0.7 ± NA	NA
No (n = 23)	27.3 ± 20.2	3.35 ± 3.46	10.4 ± 11.8
Subglotic stenosis			
Yes (n = 1)	23.5 ± NA	NA	NA
No (n = 24)	25.7 ± 20.4	3.12 ± 3.38	10.4 ± 11.8
Diabetes Mellitus			
Yes (n = 2)	12.4 ± NA	1.55 ± 2.19	21.3 ± NA
No (n = 23)	26.4 ± 20.1	3.44 ± 3.58	9.28 ± 11.8
Partial agenesis c.c.			
Yes (n = 1)	NA	NA	2.6 ± NA
No (n = 24)	25.6 ± 19.7	3.12 ± 3.38	11.2 ± 12.1
Tetralogy of Fallot			
Yes (n = 1)	NA	0	21.3 ± NA
No (n = 24)	25.6 ± 19.7	3.41 ± 3.39	9.28 ± 11.8
Adenoidectomy			
Yes (n = 22)	26.4 ± 20.1	3.13 ± 3.55	10.7 ± 12.4
No (n = 3)	12.4 ± NA	3.1 ± NA	7.5 ± NA
Tonsillectomy			
Yes (n = 20)	28.2 ± 20.7	2.41 ± 2.41	8.54 ± 5.81
No (n = 5)	14.2 ± 10.2	11 ± NA	15.3 ± 22.9
Ear Drains			
Yes (n = 19)	20.6 ± 14.8	3.3 ± 3.91	10.2 ± 13.2
No (n = 6)	40.5 ± 27.3	2.78 ± 2.45	10.8 ± 9.12

Data expressed as mean ± standard deviation and qualitative data expressed as number in the brackets. NA, Not Applicable, due to small group; ASD, Atrial Septal Defect; AVSP, Atrioventricular Septal Defect; Partial agenesis c.c., Partial agenesis corpus callosum; VSP, Ventricular Septal Defect; Adenoidectomy, with or without tonsillectomy; Tonsillectomy, with or without adenoidectomy; Ear Drains, associated to adeno/tonsillectomy.

The preoperative BMI was 18.8 ± 5.9, short-term post-surgery 19.64 ± 7.21, and long-term 23.29 ± 9.50. As regards to the respective BMI percentiles and *Z*-scores, besides the subjects’ weight classification over time, see Table 4. There is a trend in weight gain in DS population after adenotonsillectomy.

**Table 4:** BMI percentiles/Z-scores and number of subjects per weight classification over time: pre-surgery, short- and long-term post-surgery.

	Pre-surgery	Short-term post-surgery	Long-term post-surgery
BMI Percentile	69.4 ± 27.6	72.7 ± 20.7	87 ± 15.8
Z-Score	0.905 ± 1.35	0.984 ± 1.13	1.525 ± 0.89
Normal weight	10	7	4
Overweight	2	1	‒
Obesity class I	4	3	7

BMI percentile and Z-Score expressed as mean ± standard deviation. BMI, Body Mass Index. Pre-surgery, Total 18-subjects, 2-subjects were under 2-years-old, and a BMI percentile calculation was not possible. Post-surgery short-term: 11-subjects. Post-surgery long-term: 11-subjects.

## Discussion

The main finding of this study was that children with Down syndrome present a short-and long-term improvement after adeno/tonsillectomy; nevertheless, there is a significant increase in OAHI levels after 18-months of follow-up. The treatment for postoperative residual OSAS is still a challenge, and few studies address this matter. Kang, K. T. et al. concluded that, despite postoperative residual OSAS remaining in roughly half of the children, adenotonsillectomy might be effective in treating OSAS.^15^ Rayasam S. S. et al. also agree that adenotonsillectomy is an effective procedure at improving OSAS, but not at resolving, especially in children with Down syndrome, craniofacial abnormalities, GERD or pre-operative severe OSAS. Those may benefit from longitudinal post-operative PSG assessment for residual OSAS.^16^ In our clinical practice, the fact that parents reported an improvement of snoring, oral breathing, respiratory pauses and/or restless sleep was not sufficient to preclude a post-operative PSG, as it is known that even in these cases OSAS can remain.^1^^,^^5^^,^^6^ Besides the low MinO2Sat pre-surgery, 75.29 ± 12.62, there were persistent low means in short- and long-term post-surgery, 72.0 ± 16.82 and 81.61 ± 12.12, respectively. Sleep improvements based only on subjective patterns should not be considered sufficient.

Specific phenotypic features also influence the response to therapy. This study presented the increase in BMI percentile and in the obesity levels along the time in this population. Previous studies also demonstrated the association between high BMI and worsening in OAHI levels.^17^^,^^18^ Although Gouden M.R. et al. found limited evidence regarding BMI and outcome post-surgery, the authors suggested that those who were normal weight or overweight had a significant reduction in the OAHI, differently from those who were obese.^19^ What regards the recent improvement in phenotypic diagnosis, Drug Induced Sleep Endoscopy (DISE) can provide valuable information on the site(s) of obstruction, helping to differentiate primarily from secondary OSAS. Parikh et al. presented a consensus statement for the scoring of pediatric DISE in the management of pediatric OSA. The group proposed the use of a 3-point Likert scale for each anatomic site for maximal observed obstructions of <50% (Score 0, no-obstruction), ≥50% but <90% (Score 2, partial obstruction), and ≥90% (Score 3, complete obstruction). Anatomic sites to be scored during DISE that reached consensus or near-consensus were the nasal passages, adenoid pad, velum, lateral pharyngeal walls, tonsils (if present), tongue base, epiglottis, and arytenoids.^20^

As the association between OSAS, hypoxic burden and higher risks of developing cardiovascular/metabolic diseases is well known,^21^^,^^22^ these results remain relevant. Success of treatment in this population should be evaluated not only by OAHI, but also by oxygenation measurement. This study presented a significant improvement in oxygenation, with T90 decreasing from 10% to 1.7% in a long-term above 18-months. To achieve better results, this study call the attention for the correct indication of surgery, in the right moment and for well-selected DS patients. Thottam et al. presented that tonsillar-hypertrophy from Grade II and nasopharyngeal-tonsil hypertrophy occupying more than 50% of the cavum influenced a significant decrease in the central apnea index.^23^ In our surgical routine, the indication depended on the nasopharynx obstruction level (>50% obstruction), and the oropharynx obstruction level (≥Grade II). The combination of Mallampati score with tonsil-hypertrophy grade also played an important role for the joint surgical decision with the parents. Nevertheless, in most of the cases, 64% (n = 16), the combination adenotonsillectomy was the surgery of choice. It is also important to highlight that, to predict the efficiency of the surgery for this specific population, physicians should also take in account the craniofacial anatomical alterations and the BMI.^2^^,^^7^

Due to endocrine, morphologic, cardiac, genetic and lifestyle factors, the complete cure of OSAS in DS is much less achieved than the reduction of at least 50% in OAHI levels.^1^^,^^24^ In other words, the aim of the surgery is the OAHI decrease, instead of the reduction of OSAS severity. Maris et al. observed that residual OSAS was found in 60% of the 20 children submitted to adenoidectomy and/or tonsillectomy.^25^ Likewise, Merrell et al. observed the normalization of OAHI in only 52% of the 21 children with DS in PSG performed at least 6-weeks post-operatively.^26^ In our study with 25 children, 28% in a short-term and 44% in a long-term post-surgery remained with residual OSAS. The mean OAHI in a short-term was 3.12 ± 3.38 and in a long-term 10.37 ± 11.76 events/hour; although those values represent qualitatively light and severe OSAS, quantitatively they represent a significant decrease, when compared to the pre-surgery profile of severe OSAS with OAHI of 25.55 ± 19.70 events/hour. This is in line with the study of Thottam et al., which also showed that, although the OSAS profile severity stayed at the same level, the OAHI-index diminished from 26.6 to 11.6 events/hour in 18 children with DS.^27^

Kang K.T. et al. mentioned that AT for children without comorbidities is more efficient; as for Down syndrome, Prader-Willi syndrome, sickle cell disease and cerebral palsy, adenotonsillectomy is less effective and associated with more postoperative complications.^15^ In this study, laryngomalacia and ASD present the highest OAHI means, followed by AVSD. However, due to our small sample, there was no statistical significance in the association of comorbidities and OAHI/OSAS severity. In the study of Lal, C. et al., hypothyroidism lead to an increase in N1 stage of NREM sleep, representing superficial sleep, worsening in sleep-efficiency, apnea-hypopnea, and CAI.^28^ The association between central and obstructive apnea in DS patients suggests the existence of additional mechanisms contributing to central apnea pathogenesis in patients with heart disease. DaRocha et al. showed that the CAI of DS children with heart disease worsened after AT, as well as the SE, N1 sleep stage, AHI and CAI in patients with hypothyroidism.^1^ Thottam et al. also showed a postoperative reduction in the CAI in patients without heart disease, whereas the patients with the disease showed an increase in this same index.^23^ This is in accordance with our results, as this study also showed an increase of central apneas in a short- and in a long-term post-surgery. The alteration in cardiac output that can be observed in these patients can influence pulmonary gas exchange and peripheral microcirculation, generating differences in oxygen and carbon dioxide concentrations, which could contribute to the maintenance of central apnea^1^. However, both in this study and in the previous mentioned studies the values of carbon dioxide were not assessed. With regard to the paradoxical MinO_2_Sat(%) short-term values seen in our results, more studies are needed to further clarify the role of the local post-surgical edema and/or the PSG timing.

This study has some limitations due to the small sample size, which limits the generalizability, the heterogeneous sample, and the missing data, such as the lack of PSG parameters pre- or post-therapy. Data was analysed carefully, although bias can be introduced by missing PSGs and variable follow-up times. In spite of this study did not analyse the hypoventilation measurements, all the measurements and diagnostics were conducted by sleep specialists and followed the OSAS criteria. Even if the study highlighted SpO_2_ nadir at all 3-time-points, there was no supplemental oxygen delivery during polysomnography exams. The intervals between therapy and polysomnography were not standardized, which may have influenced the outcomes. As we aimed at the sleep objective parameters, there was no data over parents' subjective perception regarding their children’s sleep patterns. The results regarding only one specific type of surgery (adenotonsillectomy, tonsillectomy-alone or adenoidectomy-alone) were not described in detail due to their small samples. Although this study did not use DISE before surgery for an acurate diagnosis, it was possible to include BMI percentiles according to the weight-for-age chart and *Z*-score, which influences PSG outcomes pre- and post-surgery. More studies are needed to further clarify the correlation between BMI and persistent OSAS in this population, taking into account the anatomical bias.

In short, invasive procedures should be considered especially in children with DS with severe OSAS. However, parents should be aware of the high probability of residual OSAS in the short- and/or long-term, to create realistic expectations over the surgery. This research allows pediatric practitioners to inform the patient's family over the evolution of sleep disorders after the proposed surgery, under the influence of a variety of possible comorbidities.

## Conclusion

Adeno/tonsillectomy for children with DS and OSAS brings a significant lowering on OAHI in a short- and long-term post-surgical follow-up, although residual disease remains, with a long-term OAHI significantly higher than the short-term. More studies are needed, based on prospective multicenter standardized analysis.

## ORCID ID

Sara Costa Gomes: 0000-0001-6357-2047

Jaouad Abari: 0000-0002-5141-4292

Helen Franckx: 0000-0001-6256-9348

Marie Reynders: 0000-0003-1490-0635

Linde Peeters: 0000-0001-5031-7231

Wilfried Cools: 0000-0002-3458-0336

Ina Foulon: 0000-0002-2480-0948

## Justification for more than 7 co-authors

The therapy of children with Down Syndrome is multidisciplinary and many doctors from our institution are involved in the follow-up of these patients. Therefore, all mentioned co-authors worked actively in this research.

## Financing

No funding was secured for this study.

## Data availability statement

The authors declare that all data are available in repository.

## Data availability

The data that has been used is confidential.

Data will be made available on request.

## Declaration of competing interest

The authors declare no conflicts of interest.
